# Childhood Adversity, Recent Life Stressors and Suicidal Behavior in Chinese College Students

**DOI:** 10.1371/journal.pone.0086672

**Published:** 2014-03-28

**Authors:** Zhiqi You, Mingxi Chen, Sen Yang, Zongkui Zhou, Ping Qin

**Affiliations:** 1 Key Laboratory of Adolescent Cyberpsychology and Behavior (CCNU), Ministry of Education, Wuhan, China; 2 School of Psychology, Central China Normal University, Wuhan, China; 3 National Center for Register-based Research, University of Aarhus, Aarhus, Denmark; 4 National Center for Suicide Research and Prevention, Institute of Clinical Medicine, University of Oslo, Oslo, Norway; Radboud University, Netherlands

## Abstract

**Background:**

Although the independent effects of childhood adversities and of recent negative events on suicidality have been well-documented, the combinative role of childhood and recent adversities on risk for suicidality is still underexplored, especially in the context of Chinese culture and in consideration of specific types of negative events.

**Method:**

5989 students, randomly sampled from six universities in central China, completed the online survey for this study. Suicidal behavior, life adversity during childhood and stressful events in recent school life were assessed with designed questionnaires.

**Results:**

Students experiencing recent stressful life events more often reported an experience of life adversity during childhood. While recent stressful life events and childhood life adversity both were associated with an increased risk for suicidal behavior, the two exposures presented conjunctively and acted interactively to increase the risk. There was noticeable variation of effects associated with specific childhood life adversities, and sexual abuse, poor parental relationship, divorce of parents and loss of a parent were among the adversities associated with the highest increased risk. Recent conflicts with classmates, poor school performance and rupture of romantic relationships were the recent school life stressors associated with the highest increased risk.

**Conclusions:**

Childhood adversity and recent school life stressors had a combinative role in predicting suicidality of young people studying in Chinese colleges. Unhappy family life during childhood and recent interpersonal conflicts in school were the most important predictors of suicidality in this population.

## Introduction

Suicidal behavior in young people has become a significant public health problem and has been the focus of many studies world-wide. Consequently it is urgent to study the associated risk factors for a better understanding of this problem and to establish assessment and prevention systems. In China, suicide among young people has become a particularly serious challenge for public health. There were up to 2,000,000 Chinese attempting suicide every year, with yearly suicide casualties of 287, 000 in the period of 1995–1999 [1]. Although it is the leading cause of death for young people in China, suicide in this population has not been much investigated and relevant evidence of significant correlates is meager compared with that of western countries.

Suicidality, including suicidal ideation and suicide attempt, may be influenced by many factors, ranging from genetics [2], family functions [3], social-economic status [4], personality [5] and psychiatric comorbidity [6]. The experience of adverse events during childhood has long been known to be a significant risk factor for suicidal behaviors among young adults. A large body of research, either with clinical samples [7], community samples [8], or specific groups such as drug abusers [9] and males with hyperactivity-inattention symptoms [10], has revealed that a higher number of adverse experiences in childhood were linked with higher risks for suicidal ideation or suicide attempt. Similar results were also found in one study focusing on college students [11]. At the same time, recent negative life events confer another striking risk factor for suicidal behaviors among young people. Consistent evidence has shown that recent negative life events are associated with an increased risk for onset of suicidal ideation and suicide attempt [12], and that the number or the severity of recent life events could predict the severity of suicidality and its repetition [13].

Though childhood adversity and recent negative events are individually known as prominent risk factors for suicidal behavior, few studies have explored the combinative role of these two exposures on suicidality. According to the stressor-diathesis model of suicidal behaviors proposed by Dr van Heeringen [14], suicidal behavior should be not only influenced by the independent effects of diathesis and stress, but also further formed by their interaction. Moreover, certain diatheses (usually associated with early childhood experience and genetics) could increase the likelihood of people encountering stressful events later in life [14]. It is therefore very likely that childhood adversities and recent life stressors have a multiplicative impact on suicidality.

Available studies on childhood adversities and suicidal behavior have delved into the influence of sexual abuse and neglect, leaving the influence of many common adversities in one's early life unascertained [15]. To our awareness, in research on negative events prior to suicidal behaviors, no study has simultaneously considered types of childhood adversity and recent negative events as well as their joint effects on suicidality.

To amend the missing knowledge in the area, this study aims: a) to examine the independent association of childhood adversities and recent life stressors with suicidality in a large sample of Chinese college students; b) to explore the interactive effect of the two exposures; and c) to probe the specific impacts of different types of childhood adversities and recent school life stressors on suicidal behaviors in this population.

## Method

### Ethical approval

The study was approved by the Ethical Committee for Scientific Research at Central China Normal University. A signed consent form was collected from each student.

### Participants of Study

Of eight universities in Wuhan, a city in central China, six agreed to join in the survey. All were attached directly to the ministries of P. R. China. Stratified cluster sampling was used first to identify 10% of students at each university and then to randomly select participants from classes. Of a total 7022 students selected into the study, 6096 students participated in the survey (response rate: 86.8%). 5989 respondents completed all question items designed for the present study and therefore were considered as study population in the data analyses. The sample comprised 3156 (52.8%) male and 2768 (46.2%) female students. There were 56 respondents who did not report their gender. The age range of the sample was from 14 to 26 years old (*M* = 19.94; *SD* = 1.38).

### Instrument for assessment

#### Childhood Adversities (CA)

In this section, participants were asked to report the worst adverse experience in the first 10 years of their life. The Childhood Adversity questionnaire listed 6 adversities that children might experience: divorce of parents, poor parental relationship, loss of a parent, family financial problems, sexual abuse, and severe physical illness, together with the option of “None of the above”. Participants were asked to review each item in this part and to report if they had ever undergone any of these adversities, and if yes, then to specify which adverse experience had the worst impact on them. The score range of CA was 0–1. Students reporting “None of the above” were assigned a score with “0” and classified as “without childhood adversity”, while those reporting they had experienced one or more adversities were assigned a score of “1” and classified as cases “with childhood adversity”.

#### School Life Stressors (SLS)

To assess recent negative life events in college students, the SLS questionnaire was created to measure frustrations in different domains that college students might encounter. Based upon results from the pilot investigation for this study, nine stressors were considered: 1) poor school performance, 2) rupture of romantic relationships, 3) difficulty of adapting college life, 4) financial problems, 5) employment stress, 6) conflicts with classmates, 7) failure in making close friends and 8) other frustrations. The participants were asked which frustrations they had encountered during the past one year period. Students who reported at least one of the listed stressors were assigned a score of “1” and regarded as “with school life stressor’, while those reporting “None of the above” were assigned a score of “0” and classified as “without school life stressor”.

#### Suicidality

Participants' suicidal ideation and suicide attempts were assessed by a questionnaire comprising 5 questions: 1) Did you ever seriously consider killing yourself in the past year; 2) Have you ever seriously considered killing yourself in your life; 3) Did you ever try or attempt to kill yourself within the past one year; 4) Have you ever tried or attempted to kill yourself in your life; and 5) Have you ever had a nonfatal suicide action. Participants answered the first 4 questions by marking 0 (never), 1 (sometimes) or 2 (very often). For the last question respondents had to answer only “Yes” or “No”. Participants were asked to respond to all items. Students who answered “never” for both items about suicidal ideation (items 1 and 2) were classified as “Without suicidal ideations”, while the others were classified as “With suicidal ideations”. Similarly, students who chose “no” or “never” as the answer to all three questions about suicide attempts (items 3–5) were classified as “Without suicidal attempts”; otherwise, they were regarded as “With suicidal attempts.”

#### Demographic information

Personal demographic information was also collected during the survey. Respondents reported personal details including age, gender, specialty of study, and region of permanent family residence (urban or rural).

### Procedure

An independent website was designed for this project with all relevant questionnaires and the site was accessible only through a unique password assigned to each selected student. The students were asked to complete the survey online. Before the survey, they were given a brief introduction to the study and were ensured of the confidentiality of personal data. Students who agreed to participate signed a consent form. The on-line survey started with an overall introduction about the research purposes, and then moved to specific instructions for each questionnaire. Several pilot studies were carried out to examine whether the questionnaires were suitable and understandable, and also to test the functionality of the website. There was no report of technical problems during the final online survey collecting data from the students.

### Data Analyses

Data were first generated into a spreadsheet file and then analyzed with SPSS17.0. Firstly, a t-test was used to detect if there was any difference in the number of school life stressors according to the presence of childhood adversity. Secondly, three logistic regression models were conducted to assess the effects of gender, region, childhood adversity and school life stressors on risks for suicidal ideation and for suicide attempts. Model 1 estimated the individual effect of each of these variables; Model 2 estimated the separate effects of CA and the SLS with the adjustment for gender and region; while in Model 3 the effects of gender, region, SLS and CA on suicidality were considered simultaneously in one analytic model. Lastly, separate regression analyses were conducted to examine specific CA and SLS events as risk factors for suicidal ideation and suicide attempt.

## Results

Among the 5989 college students, 982 (16.40%) presented a positive answer to suicidal ideation some time during their life course, while 115 (1.92%) reported the presence of a suicide attempt. The prevalence of both suicidal ideation and suicide attempt was significantly higher in female students than in male students. Table 1 shows the prevalence of suicidal behaviors by variables of interest in the present study.

**Table 1:** Prevalence of suicidal ideation and suicide attempts in the study population.

Variables under study		Number	Suicidal ideation		Suicide attempts	
		N	%	(n)	%	(n)
Gender	Male	3165	13.02	(412)	1.33	(42)
	Female	2768	20.38	(564)	2.60	(72)
Region	Urban	3942	16.39	(646)	2.23	(88)
	Rural	2044	16.39	(335)	1.32	(27)
School life Stressor	No	953	6.40	(61)	0.84	(8)
	Yes	5036	18.29	(921)	2.13	(107)
Childhood adversity	No	3698	13.63	(504)	1.33	(49)
	Yes	2291	20.86	(478)	2.88	(66)
Total		5989	16.40	(982)	1.92	(115)

Note. In the study population, 56 students did not report gender information and 3 students did not report the region of permanent family residence. .

Examination of the relationship between childhood adversities and school life stressors showed that in the group of students “with childhood adversities”, the mean number of school life stressors was 2.51±1.47, whereas the corresponding number was 1.86±1.34 for the group “without childhood adversities”. The t-test result indicated that compared to those without any adversity in childhood, individuals who had one or more adverse childhood experience experienced more recent negative events in school (t = 30.28, p<0.001).

Table 2 shows the effect of study variables on risk for suicidal ideation, derived from three logistic regression models. Results from model 1 revealed that gender, recent school life stressors and childhood adversities all had a significant association with suicidal ideation, while region of family residence did not. Compared with model 1, the OR values associated with SLS and CA in Model 2 were slightly reduced after adjustments for the effects of gender and region. When all variables were simultaneously included in model 3, gender, region, SLS and CA all had a notable influence on suicidal ideation. Moreover, compared with the estimates from model 2, the OR associated with SLS decreased by 3.60% and the OR associated with CA reduced by 15.38%, suggesting that there was an interactive effect of SLS and CA on risk for suicidal ideation.

**Table 2:** Effects of gender, region, school life stressors and childhood adversities on risk for suicidal ideation and for suicide attempt.

Variables	Model I				Model II				Model III			
	Wald	*p*	OR	95%CI	Wald	*p*	OR	95%CI	Wald	*p*	OR	95%CI
**Suicidal ideation**												
Gender (0/1 = Male/Female)	57.87	0.00	1.71	1.49–1.97					60.19	0.00	1.76	1.53–2.03
Region (0/1 = Urban/Rural)	0.71	0.94	0.94	0.82–1.08					8.62	0.00	0.80	0.70–0.93
School life stressors (0/1 = No/Yes)	180.24	0.00	1.37	1.31–1.44	184.99	0.00	1.39	1.32–1.45	141.72	0.00	1.34	1.28–1.40
Childhood adversities (0/1 = No/Yes)	87.64	0.00	1.93	1.68–2.22	100.39	0.00	2.08	1.80–2.40	56.48	0.00	1.76	1.52–2.04
**Suicide attempt**												
Gender (0/1 = Male/Female)	12.26	0.00	1.99	1.35–2.92					11.24	0.00	1.94	1.32–2.86
Region (0/1 = Urban/Rural)	6.64	0.01	0.60	0.41–0.89					11.34	0.00	0.51	0.34–0.75
School life stressors (0/1 = No/Yes)	28.84	0.00	1.36	1.22–1.52	31.04	0.00	1.38	1.23–1.54	21.18	0.00	1.32	1.17–1.48
Childhood adversities (0/1 = No/Yes)	17.85	0.00	2.24	1.54–3.25	23.54	0.00	2.58	1.76–3.79	14.96	0.00	2.17	1.47–3.22

Notes: Model I: analyses without any adjustment; Model II: only adjusted for gender and region; Model III: adjusted for all variables listed in the table.

Table 2 also displayed the results of regression analyses focusing on suicide attempt. Again, the effect sizes associated with the variables under study, as well the pattern of the effects, were very similar to those found in the analyses on suicidal ideation. From model 2 to model 3, there was a 4.35% reduction of the OR value associated with SLS and a 15.89% reduction of the OR associated with CA, suggesting an interactive effect of the two exposures on risk for suicide attempt.

Analyses were then conducted to examine the associations between suicidality and each item in the CA and SLS questionnaires. Associations between each specific childhood adversity and suicidality are shown in Table 3. Family financial problems constituted the most common childhood negative event (21.8%) for the Chinese college students, followed by poor parental relationship (9.5%). Among the childhood adverse experiences, sexual abuse, divorce of parents, poor parental relationship and loss of a parent were associated with significantly increased risks for suicidal ideation (*p*<0.01 for all) and suicide attempt (*p*<0.01 for all). Though financial problems and severe physical illness in childhood had a significant effect on suicidal ideation (*p*<0.01), they did not have a significant influence on suicidal attempt (*p* = 0.20 and *p* = 0.26, respectively). Among all childhood adversities under study, the three carrying the most risk for suicidal ideation were sexual abuse, poor parental relationship and loss of a parent; for suicide attempts, the top three risk factors were sexual abuse, loss of a parent and divorce of parents. It is worth mentioning that even with the limited number of college students who reported sexual abuse, this form of childhood adversity carried the highest risk for suicidality.

**Table 3:** Logistic regression results showing risk of suicidal behaviors associated with specific adverse experiences in childhood.

			Suicidal ideation				Suicide attempts			
		cases	Wald	*p*	OR	95%CI	Wald	*p*	OR	95%CI
STEP 1	Gender		53.25	0.00	1.70	1.48–1.96	1.24	0.27	1.19	0.88–1.60
	Region		2.10	0.15	0.90	0.78–1.04	4.26	0.04	0.72	0.53–0.98
STEP 2	Divorce of parents	140	13.92	0.00	2.16	1.44–3.28	18.71	0.00	4.06	2.15–7.66
	Poor parental relationship	566	95.58	0.00	2.83	2.30–3.49	47.37	0.00	3.86	2.63–5.68
	Loss of parent(s)	110	17.92	0.00	2.62	1.68–4.10	16.20	0.00	4.39	2.14–9.02
	Financial problems	1307	28.05	0.00	1.62	1.36–1.94	1.64	0.20	1.32	0.86–2.01
	Sexual abuse	19	9.83	0.00	4.36	1.74–10.98	11.04	0.00	8.45	2.40–29.78
	Severe physical illness	149	19.4	0.00	2.44	1.64–3.62	1.30	0.26	1.71	0.68–4.29

Note. ORs were adjusted for all childhood adversities as well as demographic variables listed in the table.

Table 4 shows the relationships between each specific stressor on the SLS and suicidal behaviors in the study population. Poor school performance was the most common stressor in college life, presenting in 56.44% of the study population. Poor school performance, rupture of a romantic relationship, failure to adapt, financial problems, conflicts with classmates, failure to make close friends, and other frustrations, all had a significant effect (*p*<0.01) on suicidal ideation. For suicide attempts, only poor school performance (*p*<0.05), rupture of romantic relationships (*p*<0.01), failure in adaptation (*p*<0.01) and financial problems (*p*<0.05) had a significant influence. Conflicts with classmates, poor school performance and other frustrations carried the highest risk for suicidal ideation, while rupture of romantic relationships, difficulty in adaptation, and financial problems were carried the highest risk for suicidal attempt. Furthermore, there was no significant effect of stress from employment competition on suicidal behavior in the study population.

**Table 4:** Logistic regression results showing risk of suicidal behaviors associated with specific stressors in school life.

			Suicidal ideations				Suicidal attempts			
		case	Wald	*p*	OR	95%CI	Wald	*p*	OR	95%CI
STEP 1	Gender		56.72	0.00	1.74	1.51–2.01	3.35	0.06	1.32	0.98–1.79
	Region		2.07	0.15	0.90	0.80–1.04	6.15	0.01	0.68	0.50–0.92
STEP 2	Poor school performance	3380	28.15	0.00	1.50	1.29–1.74	4.40	0.04	1.41	1.02–1.93
	Rupture of love affairs	1269	9.30	0.00	1.30	1.10–1.53	9.00	0.00	1.65	1.19–2.29
	Difficulty in adapting college life	1545	21.91	0.00	1.45	1.24–1.69	8.89	0.00	1.62	1.18–2.22
	Financial problems	1229	12.52	0.00	1.36	1.15–1.61	4.54	0.03	1.45	1.03–2.04
	Employment stress	1176	0.10	0.75	0.97	0.81–1.16	0.61	0.43	1.15	0.81–1.64
	Conflicts with classmates	706	21.09	0.00	1.59	1.30–1.93	3.03	0.08	1.42	0.96–2.10
	Failure in making close friends	2011	14.68	0.00	1.34	1.15–1.56	1.75	0.18	1.24	0.90–1.70
	Other frustration	1516	26.40	0.00	1.49	1.28–1.74	0.34	0.56	1.10	0.79–1.53

Note. ORs were adjusted for all school life stressors as well as demographic variables listed in the table.

## Discussion

### Demographic structure of suicidality in Chinese college students

The present study demonstrates that both suicidal ideation and suicide attempts were significantly more common in female students as compared with their male counterparts. This observation is consistent with the conclusion of a previous study on Chinese college students [16], and also in high concordance with many reports internationally [17]–[19]. As for the effect of region, there was no significant rural-urban difference for suicidal ideation, but the risk of suicide attempt was significantly higher among students coming from urban areas than those from rural China. This finding is somewhat different from the previous view that the suicide rate in rural China is much higher than that in urban places [1], [20]. A possible reason could be that the sample population in the current study is very different from those in previous reports. College students coming from rural areas were now studying and hence living in a city of central China. They were pursuing an education and enjoying a much better living environment than what they had in the rural area. It is therefore understandable that these students are at a relatively lower risk for attempting suicide compared with counterpart students coming from the urban areas.

### Childhood adversity, school life stressors and suicidality

This study shows that both adverse experiences in childhood and recent life stressors in school life are strongly associated with risk for suicidal behaviors in young people studying in university. These results are consistent with previous reports focusing on early life adversities [7], [8], and also studies on recent stressful life events in relation to suicidality [12], [13].

This study adds to the literature demonstrating that experience of childhood adversity (CA) could increase the likelihood of exposure to negative life events (SLS) during young adulthood and that there is an interactive effect between SLS and CA on suicidality. Such observations support the stress-diathesis model of suicidal behaviors, proposed by Dr van Heeringen [14]. Apart from the respectively independent effect of school life stressors and childhood adversities on suicidality, the two types of exposure combined to exert an interactive role on suicidality. According to the stress-diathesis model, childhood adversities could act as a diathesis that could predispose individuals to more negative events in their future life; the occurrence of later stressful events could in turn further predispose these people to become more vulnerable to the effects of stressful events; and consequently, risk for suicidal behavior becomes higher and higher through an interactive process. There are a number of published studies providing evidence of the proposed impact pathway. For example, childhood adversity was found to be linked with personality characteristics such as aggression; the aggressiveness made the subjects more maladapted and therefore induced more stressful events [21]. Childhood adversity could increase the tendency to form specific cognitive characteristics, such as negative attribution style [22] and hopelessness [23], which could in turn increase the vulnerability to new stressors.

The observed interaction between childhood adversity and recent school life stressors could also be interpreted from developmental perspectives. One possible explanation lies in the association between attachment and suicidality. Secure attachment has been acknowledged as a strong, if not the most important, protective factor for children's psycho-social development. Especially in the face of troubles or frustrations, secure attachment could be the child's major psychological resource [24]. As well established, early adversities or traumas are detrimental for the formation of secure attachment [25].Therefore, children with severe childhood adversity might show less secure attachment during their upbringing and show greater vulnerability in response to stressful events compared to peer youth raised in families that experienced no obvious adversity. It is also more difficult for them to retain stable and durable relationship with others, including with family members and classmates [26] – factors strongly associated with suicidality among young people [27]. The current results are consistent with the attachment hypothesis, in that interpersonal stress and conflicts with classmates showed high prevalence among students with suicidal behavior.

Moreover, childhood adversity and recent life stressors may share certain characteristics as risk factors for suicidality, the most notable being difficult family relationships. Family circumstances are usually enduring and related both socially and psychologically to the adjustment of individual family members. Children from such families may experience ongoing suffering and are more likely to experience stressors from childhood through adolescence to adulthood. This is supported by early research demonstrating strong associations between a problematic family background, early life adversity and recent stressful life events [28]. It is also evident that children from family backgrounds characterized by low socio-economic status, poor communication or troublesome life are at an increased risk for suicidal behaviors [24].

### Influence of specific life stressors or negative events

The present study indicates that different types of life stressors or negative events, either in childhood or in school life, had different effects on suicidal behavior in Chinese college students. Compared with other stressful events, poor school performance was the most common stressor that Chinese college students encountered within a one year period. Among all stressors under study, those that were significantly associated with suicidality (both suicidal ideation and suicide attempts) were poor school performance, rupture of romantic relationships, difficulty in adapting school life and financial problems; conflicts with classmates, failure to make close friends and other frustrations were linked with suicidal ideation but not suicide attempts. These findings are mostly in agreement with another study of college students, showing that suicidality was especially associated with interpersonal affairs, such as interpersonal conflicts and ruptured relationships as well as school performance [29].

It is worth noting that employment stress did not confer significant risk for suicidal behavior in the present study. In earlier research the association between unemployment and suicidality has been viewed as ambiguous and conclusions have varied under different research conditions [27]. To our awareness, however, no study has focused on suicidal behavior in relation to stress from possible employment competition, let alone in a sample of Chinese college students. Yet we could not simply conclude from the present data that employment stress has no significant effect on suicidal behaviors in Chinese undergraduates, because the present sample mostly consisted of students who had not yet completed their third year at their universities. Due to their limited experiences in the labor market, it is likely that job competition is not yet an urgent consideration for the subjects of the present study. On the other hand, our data demonstrate that recent stress from financial problems is strongly associated with risk for both suicidal ideation and suicide attempt. This is in line with previous studies of young adults [30], [31], and underlines the importance of financial support for college students and of students' proper management of their economic situation during school life.

Regarding childhood adversities, it is interesting to see that all parent-related stressors during childhood –including divorce of parents, poor parental relationship and loss of a parent– were associated with a significantly increased risk for suicidal behavior. This finding is in line with the literature [17], [31], and makes particular sense when thinking of the stress-diathesis model and attachment theory discussed in the previous section. Although very few students reported the experience of sexual abuse during childhood, this exposure denoted the highest risk for suicidal ideation and attempt. On the other hand, the experience of family financial problems during childhood was the most frequently reported childhood stressor in this population, although compared to other negative childhood experiences it had the lowest influence on suicidality. Of course, family financial problems during childhood do not necessarily mean low economic status, because the problems could be due to a short term crisis. Nevertheless, the results from this study indicate that compared with insufficient resources caused by financial problems, family stressors such as parental divorce or a poor parent-child relationship play a more important role in the development of suicidality.

### Limitations

There are several limitations in the present study. One limitation is that the study is a cross-sectional investigation, which does not allow detection of any causal relationship between exposure to adversity and suicidality. It would be ideal to test the findings from this study using a cohort design, following children from their early life. Another limitation concerns generalizability. The sample was obtained strictly by a stratified cluster sampling method in six of eight universities in a major city in Central China. Although we are confident that our sample represents well the undergraduate college students in the area of Central China, it is uncertain to us if it could represent undergraduates in other places of China such as the more developed coastal or the less developed western regions. It is therefore preferable that more studies should be carried out in larger and more diverse samples to test the present findings and evaluate their generalizability.

## Conclusion

Based on a large sample of undergraduate students in China, the present study reports a 16.40% rate of suicidal ideation and a 1.92% rate of suicide attempts. The prevalence differed significantly by gender and region of family residence, with higher rates in females than males and lower rates among students from rural areas compared to urban areas. At the same time, students with suicidal behavior more often reported the experience of negative life stressors in both childhood and recent school life. While childhood adversity and recent school life stressors each denoted significant risk for suicidal behavior, the two types of exposure tended to co-occur and act interactively to increase the risk for suicidal behavior. With noticeable variation of effects associated with specific stressors, family relationship problems during childhood and recent interpersonal conflicts in school are of great importance in predicting risk for suicidality. These findings should be taken into account when planning programs of mental health promotion, suicide prevention and suicide intervention in university settings in China, and perhaps internationally.
