# Correction: Childhood Adversity, Recent Life Stressors and Suicidal Behavior in Chinese College Students

**DOI:** 10.1371/journal.pone.0101612

**Published:** 2014-06-27

**Authors:** The PLOS ONE Staff

The second author’s name is spelled incorrectly. The correct name is: Mingxi Cheng. The correct citation is: You Z, Cheng M, Yang S, Zhou Z, Qin P (2014) Childhood Adversity, Recent Life Stressors and Suicidal Behavior in Chinese College Students. PLoS ONE 9(3): e86672. doi:10.1371/journal.pone.0086672
